# Ultrasound imaging in women's arm flexor muscles: intra-rater reliability of muscle thickness and echo intensity

**DOI:** 10.1590/bjpt-rbf.2014.0186

**Published:** 2016-09-22

**Authors:** Amilton Vieira, Angelina F. Siqueira, João B. Ferreira-Junior, Paulo Pereira, Dale Wagner, Martim Bottaro

**Affiliations:** 1 Laboratório de Treinamento de Força, Faculdade de Educação Física, Universidade de Brasília (UnB), Brasília, DF, Brazil; 2 Health, Physical Education and Recreation Department (HPER), Utah State University (USU), Logan, Utah, USA

**Keywords:** ultrasonography, rehabilitation, muscle hypertrophy, greyscale analysis, biceps brachii, elbow

## Abstract

**Background:**

Different ultrasound parameters have been frequently used to assess changes associated with training, aging, immobilization, and neuromuscular diseases. However, an exploratory reliability analysis of the echo intensity (EI) and muscle thickness (MT) of the forearm flexors is scarce, especially in women.

**Objective:**

The purpose of the present study was to determine the intra-rater reliability of MT and EI assessed by ultrasound in young women.

**Method:**

Ultrasonographic MT and EI were acquired in the forearm flexors of 41 young women (22±2 yrs). Reliability was calculated using intraclass correlation coefficient (ICC_2,1_), standard error of measurement (SEM), coefficient of variation (CV), smallest detectable change (SDC), and Bland and Altman plot analysis.

**Results:**

ICC values for MT and EI were 0.88 (95% CI: 0.78-0.93). The SEM and CV values were lower than 10%. Bland and Altman analysis revealed that ultrasound mean differences were 0.27 mm (Limits of Agreement - LOA 95%: - 2.6 to 3.2 mm) and -0.09 a.u. (LOA 95%: - 10.9 to 10.7 a.u.).

**Conclusion:**

MT and EI assessed by ultrasonography in young women appear to be reliable and may be used to monitor changes in muscle mass induced by strength training when these changes exceed the precision of ultrasound.

## BULLET POINTS

• US may be used to monitor changes in muscle mass induced by exercise programs. • US may be used to monitor changes in muscle quality induced by rehabilitation. • The measurement error associated with US must be considered in the interpretation of the results. • Lower MT was associated with higher echogenicity.

## Introduction

The measurement of muscle size and morphology has been frequently used to monitor the effects of strength training, aging, and immobilization in patients with neuromuscular diseases^1^ ^-^ ^7^. Magnetic resonance imaging (MRI) and computerized tomography are considered “gold standard” devices for muscle size, morphology, and composition assessment. However, these devices are costly and typically unavailable in sports training facilities and clinical settings^8^. Thus, Brightness (B)-mode ultrasound (US) has been a good alternative to visualize normal and pathological skeletal muscle changes^9^. Bemben^10^ also highlighted that US measures are safe, quick, and more cost effective than other imaging techniques. However, care must be taken due to a number of potential measurement errors. Changes at the site where measurement is performed and probe compression rate may significantly affect US results. Consequently, studies have been conducted to validate the US measurements of muscle cross-sectional area and to determine their test-retest reliability^8^ ^,^ ^9^ ^,^ ^11^ ^,^ ^12^. For example, Reeves et al.^8^ reported that the validity of US against MRI in assessing muscle size produced excellent intraclass correlation coefficient values ranging between 0.998 and 0.999.

Since the first study using US to measure muscle cross-sectional areas^13^, its use in research, sports, and clinical facilities has grown in popularity. Currently, other US parameters have been added to the muscle unit investigation. Muscle echo intensity (EI) has attracted attention as a method of non-invasive investigation of tissue composition because it can identify fat and fibrous tissue infiltration^14^. Indeed, it has been associated with physical fitness, muscle damage, and overall muscle quality^2^ ^,^ ^4^. In addition, muscle thickness (MT) has been frequently used to assess muscle damage induced by exercise and monitor resistance training interventions on hypertrophy outcomes^6^ ^,^ ^15^.

English et al.^16^ published a systematic review that stressed that most of the reliability studies published on US variables lacked an adequate statistical analysis and a blinded rater, and these factors could lead to a large source of bias. Thus, a study using a more robust statistical approach including limits of agreement, larger sample size, and blinded raters are required^17^ ^,^ ^18^. Furthermore, according to Atkinson and Nevill^17^, US should be reliable enough to be used in a specific population. Gender differences seem to be particularly important during MT and EI assessment since it has been reported that women present thinner muscles and higher echogenicity than men over a number of muscles, such as biceps brachii, quadriceps femoris, sternocleidomastoid, tibialis anterior, and others^14^. These sex-related differences might increase MT and EI variability in women^19^. Thus, data from other populations, such as men, may not be applicable to young women. Data from the present study will be valuable for future studies to estimate sample size and to assess better the forearm flexor MT and EI adaptations in response to treatment or training in this population. Therefore, the aim of this study was to determine the intra-rater reliability of US measurements of MT and EI in the forearm flexors of healthy young women. In addition, we investigated the relationship between MT and EI since thicker muscles may also demonstrate lower echogenicity in young and healthy populations.

## Method

### Study design

A test-retest design was used to assess the reliability of MT assessment of the forearm flexor muscles and the EI of the biceps brachii. Separated by 24–48 hours, each subject was assessed twice at the same time of day by the same blinded examiner. The examiner captured the image for subsequent analysis. In order to reduce potential bias and to blind the examiner, no MT or EI measurements were taken during image capture. Later, all image measurements and statistical analyses were performed by a second, blind researcher using non-sequential numbers. The choice of these muscle groups and the US parameters were based on the large amount of experimental studies investigating these muscle groups^3^ ^,^ ^5^ ^,^ ^6^ ^,^ ^20^ ^,^ ^21^.

### Participants

The required number of subjects “a priori” was based on a tabulated chart provided by Walter et al.^22^ Considering the follow settings p0=0.60, p1=0.80, α=0.05, and β=0.20, a minimum sample size of 39 participants was needed. Although 39 subjects were technically sufficient to meet the power required, we decided to recruit 50 subjects to allow for some missing data or dropouts. A sample of 50 healthy women from a university population volunteered to participate. To be included in the study, participants had to be healthy and between the ages of 18 and 30 years. In addition, they were free from neuromuscular diseases or musculoskeletal injuries involving the upper limbs.

Participants were not allowed to perform any vigorous physical activities or unaccustomed exercise, take medications, or consume any type of supplements during the experimental period. Informed consent was obtained before testing, and the investigation was approved by the Ethics Committee of Universidade de Brasília (UnB), Brasília, DF, Brazil (approval number 788.65/14).

### Procedures

**Figure 1:**
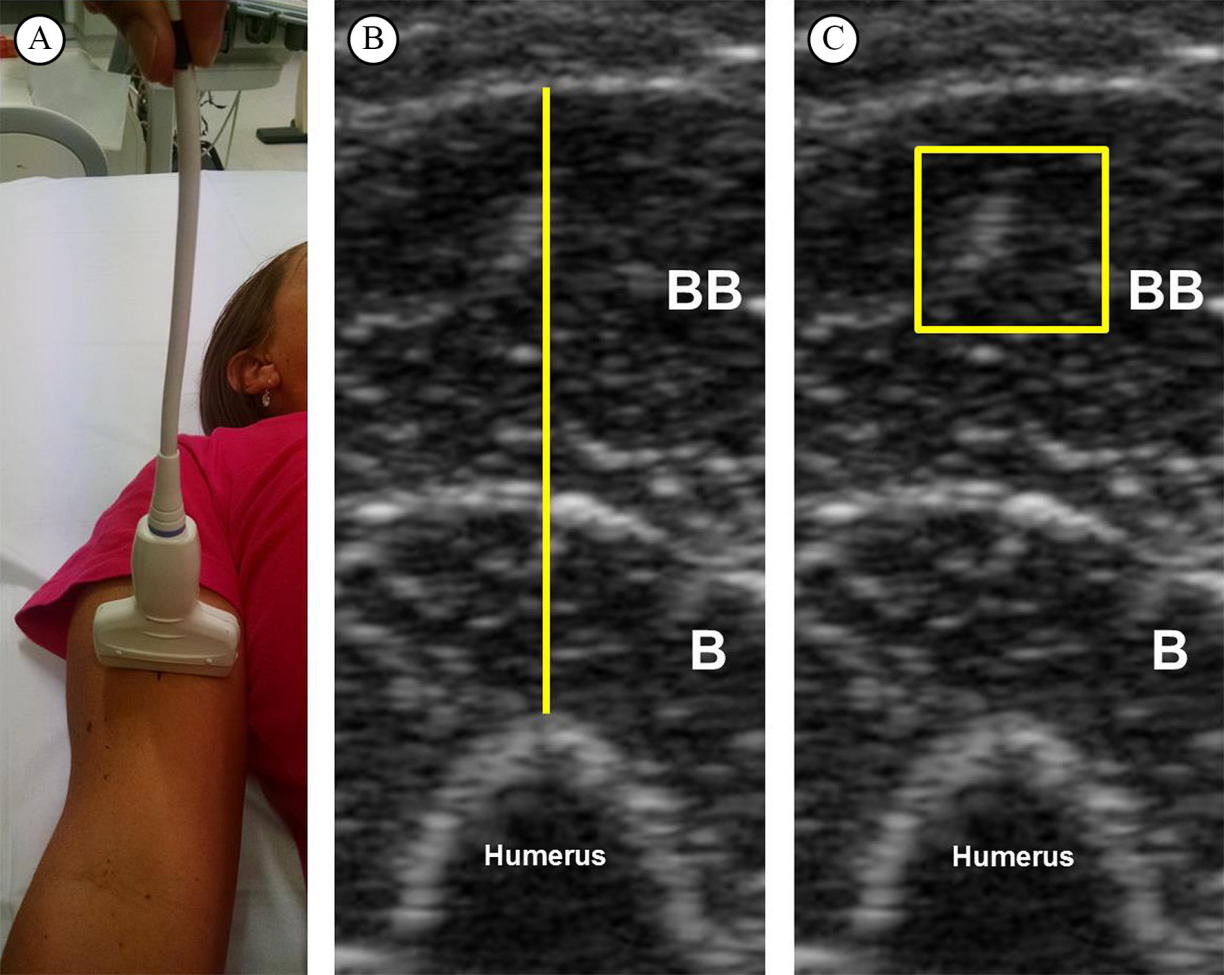
Representative image from one subject. (A) Ultrasound scan of forearm flexor muscles. (B) Muscle thickness of forearm flexors, defined as the distance between adipose tissue and bone. (C) Echo intensity of biceps brachii muscle in a square set at 100 mm^2^. BB: biceps brachii; B: brachialis.

Body height and weight were assessed using a stadiometer (Sanny, Murrhardt, Germany) and physician’s scale (Lider, São Paulo, Brazil). US B-mode scans (Philips-VMI, Ultra Vision Flip, Model BF, Minas Gerais, Brazil) from forearm flexor muscles were taken 10 cm superior to the antecubital crease^23^. This landmark was found to improve reliability^10^. The participants were evaluated in supine position with a 7.5 MHz scanning head placed on the skin perpendicular to the tissue interface^2^ ^,^ ^4^ ^,^ ^6^ ^,^ ^15^. They were asked to relax their limbs during assessment and a suitable amount of water-soluble transmission gel was used to ensure optimal image quality. To minimize the transducer pressure on the skin, it was held by a guide mark placed on the transducer’s cable (Figure 1A). For the best representation of the bone boundary, the optimal angle was selected for each scan. An exercise and health science specialist with six months of experience in skeletal muscle US imaging performed all assessments.

### Measurement of MT and EI

The images were analyzed in the software Image-J (National Institute of Health, USA, version 1.47). Initially, the subcutaneous adipose tissue–muscle interface and the muscle–bone interface were identified. Then, the distance between adipose tissue and bone was defined as MT (Figure 1B). The EI was determined by gray-scale analysis using the standard histogram function expressed by values between 0 and 256 (0: black; 256: white) for the region of interest (ROI) (100 mm^2^)^9^ ^,^ ^24^. During EI measurements, the depth of the ROI was set at 5 mm below the fascia of the biceps brachii^2^ (Figure 1C).

### Statistical analysis

To examine intersession (intra-rater) reliability, the means and standard deviations (SD) for US parameters from evaluations 1 and 2 were calculated. A dependent t-test was used to assess systematic error with the level of significance at *p*<0.05. Relative reliability was assessed by intraclass correlation coefficient (ICC). Then, standard error of the measurement (SEM), smallest detectable change (SDC), coefficient of variation (CV), and Bland and Altman plot analysis were used to assess absolute reliability^17^. ICC type 2,1 (ICC_2,1_) was used^12^ ^,^ ^19^ ^,^ ^25^. Subsequently, SEM was calculated using the following equation^17^: SEM = SD $\sqrt{1-ICC}$. SEM enabled to calculate both SDC and CV^17^ ^,^ ^25^: SDC = SEM×1.96×$\sqrt{2}$. The CV was calculated using the equation: CV = SEM×Mean^-1^. The limits of agreement (LOA) were determined based on Bland and Altman plot analysis. The absolute differences against the individual means of the two measurements were plotted in order to verify homoscedasticity. The 95% limits of agreements (LOAs) were calculated as follows: the standard deviation of the differences between evaluations 1 and 2 was calculated and multiplied by +1.96 and –1.96 to obtain upper and lower limits. Pearson product moment (*r*) was used to investigate the level of association between MT and EI. Microsoft Excel and Statistical Package for the Social Sciences (SPSS, version 17.0) were used for analysis.

## RESULTS

Fifty healthy women were recruited to participate in this study. Nine subjects did not return on the second day. Then, 41 healthy college-aged women aged 21.1±2.3 years, weight of 59.9±10.2 kg, height 162.6±7.1 cm, and body mass index of 22.6±3.3 m×m^-2^ were assessed and included in the further analysis.

**Figure 2:**
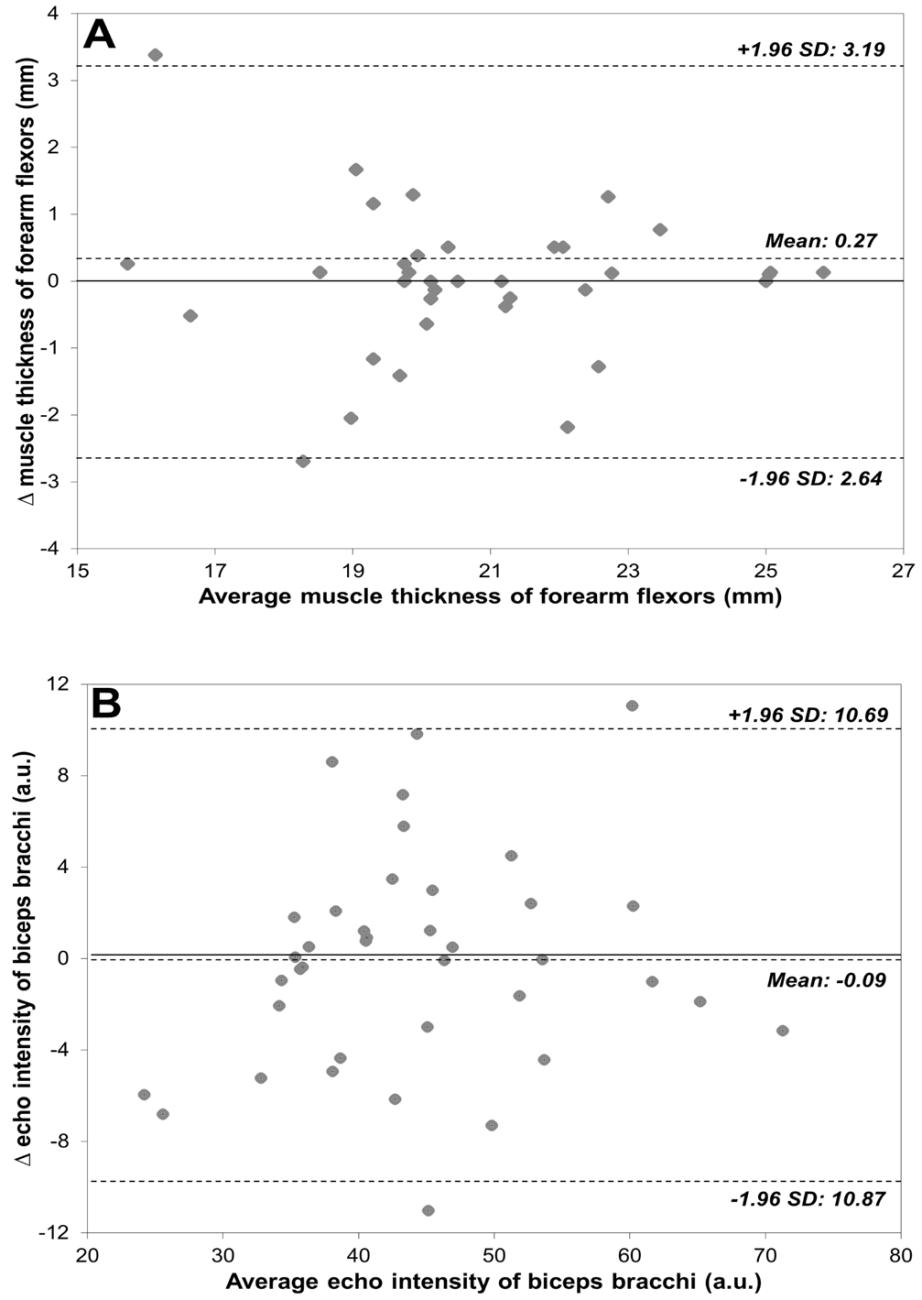
Bland-Altman plots illustrating the differences between evaluations 1 and 2. (A) Muscle thickness of forearm flexors; (B) echo intensity of biceps brachii. The bias line and random error lines forming the 95% limits of agreement are presented by dashed lines. SD: standard deviation.

**Figure 3:**
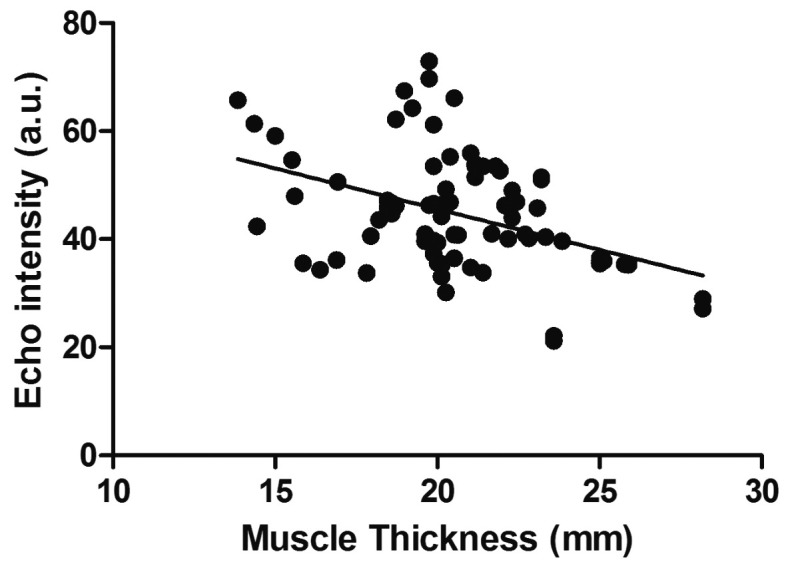
Relationship between muscle thickness and echo intensity in women (r=0.416, p<.0001).

Table 1 and Figure 2 show the reliability results for the MT and EI assessments. The ICC values were 0.88 for both MT and EI and they were classified as very good, where values ≤0.20 are considered poor, 0.21 to 0.40 fair, 0.41 to 0.60 moderate, 0.61 to 0.80 good, and 0.81 to 1.00 very good^26^. Moreover, SEM and CV values were <10%. Relative systematic error (bias) was formally evaluated using a dependent t-test across the two trials, and no bias was observed (p>0.05). In addition, SDC compromised 13.7% and 22.9% of the mean MT and EI, respectively. Bland and Altman analysis revealed that US MT mean difference of 1.3% (LOA 95%: -15.6 to 12.9) and EI of 0.2% (LOA 95%: -23.9 to 24.3). There was a moderate and negative correlation between MT and EI in women aged 18 to 28 years (r=.416, p<.0001), where 0.1 to 0.35 = weak; 0.36 to 0.67 = moderate; ≥0.68 = strong)^27^. Thus, MT statistically explained 17% (r^2^, equal to 0.416 = 0.17) of the variability in EI (Figure 3).

**Table 1:** Reliability of ultrasound analysis of the forearm flexors of women (n=41).

**Ultrasound Parameters**	**Evaluation 1**	**Evaluation 2**	**ICC_2,1_**	**SEM**	**CV**	**SRD**	**p Value**
Muscle Thickness (mm)	20.51 (4.23)	20.78 (4.42)	0.88 (95% CI: 0.78-0.93)	1.02	4.95	2.82	0.24
Echo Intensity (U.A.)	44.71 (12.70)	44.50 (12.58)	0.88 (95% CI: 0.78-0.93)	3.70	8.28	10.23	0.80

Data from Evaluations 1 and 2 are reported as mean (SD). ICC: intraclass correlation coefficient; CI: 95% confidence interval; SEM: standard error of measurement; CV: coefficient of variation; SRD: smallest real difference; p value: probability value for the t test.

## DISCUSSION

The aim of this study was to investigate the intra-tester reliability of two US parameters (MT and EI) on forearm flexor muscles in women. Our data suggest the intra-rater reliability of these US parameters is good, especially for MT. The high ICCs (0.88) and low CV and SEM values (<10%) were comparable to the reliability of previous US studies examining MT of quadriceps muscles in young men and elderly women^3^ ^-^ ^5^. This study was designed to assess reliability in young women, including a high number of subjects and using a more robust statistical approach. Data from the present study can be used to design future studies in the estimation of sample size and to assess better the forearm flexor MT and EI adaptations in response to treatment or training in this population. For example, a clinician may consider that an increase in MT following an injury-rehabilitation program should exceed the range of -2.64 to 3.19 mm for MT and -10.87 to 10.69 for EI in young women. LOA and SDC provide further insight since they represent 95% of the error related to repeated measurements instead of the ~68% reported by most methods of calculating SEM and CV^17^. Interestingly, we also observed a negative relationship between MT and EI. It may indicate that thinner muscles are associated with higher echogenicity, which has been associated with lower muscle quality^2^ ^,^ ^9^. In fact, increased echogenicity is typically observed in some myopathies and associated with aging^7^ ^,^ ^9^. Future studies are needed to examine the relationship between higher echogenicity and conjunctive tissue, subcutaneous fat, and inflammatory cell infiltration in muscular tissue.

In the exercise science literature, there is a lack of consensus on the best method for assessing reliability^17^ ^,^ ^28^. Methods based on correlation coefficients and regression (i.e. ICC) provide relative reliability, while methods expressing error in the actual unit of measurement (i.e., SEM, CV, SDC, and LOA) provide absolute reliability. These methods have strengths and weaknesses, which taken together suggest that they could complement each other. For instance, the utility of the SEM has been criticized as a measure of reliability, but it can be used to calculate the SDC. SDC is the minimal difference to be considered real and not merely measurement error. Our SDC results suggest that changes of 2.82 mm (13.7%) and 10.23 (22.9%) a.u. are the minimum values required to be considered real for MT and EI, respectively. These values can be taken into account when comparing the effect of an intervention program on MT and EI in young women. Furthermore, when more than a single method is reported in a reliability study, the reader can interpret and use the one with which they are most familiar^17^. The inclusion of the LOA method in all reliability analysis has been highly encouraged^17^. The LOA method provides the amount of measurement error in both negative and positive directions. Based on the results of the present study, further studies using these US parameters would expect (with 95% probability) that the difference between any two tests performed in a similar population should lie within the LOA presented in Figure 2. For example, for the MT of the forearm flexors of young women, it could be expected that the differences between two repeated measurements will range from -2.64 to 3.19 mm. It can also be said that, for college-aged women, two MT evaluations will differ due to measurement error by no more than 12.9% in the negative and 15.6% in the positive direction. It should be noted that the amount of error is unequal, here being greater in the positive than in the negative direction. A previous study^29^ examining the MT of trapezius muscles of 12 men and four women reported LOA ranging from -42.85% to 17.85%. These results strongly demonstrate the importance of quantifying error in both negative and positive directions. Lastly, we could conclude that B-mode US is probably not reliable enough to monitor the small changes in MT that result from increasing the training of an already athletic population, but it may detect the large differences in MT that usually follow injury-rehabilitation programs or monitor training effects in previously sedentary participants. Ultimately, it is the task of the researcher to judge whether the LOA are narrow enough for the measurement to be done or whether modifications in the experiment’s design are needed^17^.

Despite the frequent use of US devices in sports, rehabilitation, physical therapy, athletic training, and medicine research, few studies have demonstrated a proper reliability analysis in women. To the best of our knowledge, there is only a single study examining the reliability of EI in the biceps brachii of women (n=10)^9^. Although this study had shown good reliability for EI in a large range of regions of interest, they only carried out ICC and CV analysis in 10 women. Other studies^3^ ^-^ ^5^ have demonstrated high ICC (>0.90) and low CV (<5%), but they were conducted as part of a larger study in which reliability was not the primary aim and potential sources of bias might not have been adequately controlled (i.e., measures by blind rater).

The overall reliability (relative and absolute) shown in the present study seems to be worse than those reported by Jenkins et al.^12^, who used panoramic and transverse US imaging to measure similar parameters. These authors investigated the test–retest reliability and sensitivity to change for MT and EI measurements of the forearm flexor muscle in 14 men. ICC, CV, and SRD for MT and EI ranged from 0.78 to 0.99, 2.26% to 3.29%, and 6.26% to 9.12%, respectively. This discrepancy may be the result of sex-related differences in muscle morphology and composition and/or related to US settings (i.e., frequency: 7.5 versus 10 MHz and/or size of region of interest: EI 100 mm^2^ versus maximal possible). Potential reasons to explain better results in men than women include greater body mass and height, which may account for a significant portion of the variance in muscle size, and higher muscle quality often observed in men (i.e., less intramuscular fat). In view of this, Palmer et al.^19^ recently demonstrated higher EI ICCs values in men when compared to women. In fact, previous studies have reported significantly lower EI values in men versus women^14^ ^,^ ^19^. This difference may be the result of intramuscular fat and/or fibrous tissue content. It has been shown that intramuscular fat and fibrous tissue content influences EI, which also may be related to overall body fat content. Indeed, a significant positive relationship between subcutaneous fat and EI was recently observed^9^. Thus, given the difference in muscle size and quality between genders, it is prudent to have sex-specific reliability data for MT and EI. Such data will also aid in sample size estimation and interpretation of US results.

Despite considerable advantages conferred to B-mode US for muscle morphology analysis, care should be taken during both data collecting and analysis. One of the main concerns during data collection is the amount of pressure on the skin, which could deform the underlying tissues and alter the measurements. To the best of our knowledge, there is no standardized procedure to minimise this source of error; however, we believe that the procedure applied in the present study (Figure 1A) may be a good strategy to reduce the transducer pressure on the skin. Another source of measurement error could be related to acute fluid shifts in response to transition from upright to supine body position^30^. Rest periods between 15 and 20 min before each measurement have been used to allow fluid shifts to occur^1^ ^,^ ^8^ ^,^ ^31^. It is important to note that these studies were limited to the lower body, and data from Berg et al.^30^ do not support resting periods less than 30 min. Also, based on Berg et al.^30^, it seems that calf muscles are mainly affected by postural change. Our data suggest that for arm evaluations resting periods may be unnecessary for a good reliability measurement.

A possible limitation of this study was that, for repeated measurements, the US transducer was placed on the exact site (marked) on the skin. Marking the site is useful to investigate acute changes in the muscle^15^ ^,^ ^20^ ^,^ ^21^; however, for longitudinal studies the US site must be re-measured. Furthermore, considering that both MT and EI may be affected by the aging process, the findings of the present study can only be applied to young and healthy women. In addition, in the present study, we adopted a standard site (10 cm superior to the antecubital crease) to measure MT and EI. Even though, this procedure is suggested to improve within-individual reliability^10^, we might have introduced a bias due to anthropometric variations. Finally, it is also worth noting the use of a 7.5 MHz transducer. We assume that lower or higher frequencies might provide different results. The choice of frequency will be dependent on the depth of the region of interest. Usually, higher frequencies (greater than 7.5 MHz) are suggested for superficial muscles and lower frequencies (lower than 5 MHz) for deeper muscles^32^. A better detail resolution could be reflected in greater reliability especially for echo intensity analysis, which depends on image quality.

In summary, we conclude that US imaging of the MT and EI of the elbow flexors muscles of women is reliable within approximately ±3 mm for MT and ±10 a.u. for EI. The amount of error reported here should be considered when calculating sample size estimations, especially when the expected training effect is small, as in an athletic or clinical population. In addition, since the adaptations in muscle mass that occur in response to short-term strength training are small, researchers must be cautious when assessing MT and EI during the early-phase of training in this population. We also found a negative correlation between muscle MT and EI, which suggests that intramuscular fat or fibrous tissue content may exert some influence on muscle size in young, healthy women.
